# Correlations between amyloid-β peptide levels in aqueous humor and retinal thickness in patients with glaucoma

**DOI:** 10.1038/s41598-025-33346-3

**Published:** 2025-12-22

**Authors:** Tsutomu Ohashi, Takayuki Harada, Kazuhiko Namekata, Yasuhiro Shinmei, Akio Fujiya, Maiko Yoshida, Takashi Kojima

**Affiliations:** 1 Ohashi Eye Center, Kita 1-1 Hondori 6, Shiroishi, Sapporo, 003- 0027 Hokkaido Japan; 2 Visual Research Project, Tokyo Metropolitan Institute of Medical Science, Tokyo, Japan; 3 Department of Ophthalmology, Caress Memorial Hospital, Sapporo, Japan; 4 Nagoya Eye Clinic, Nagoya, Japan

**Keywords:** Glaucoma, Pseudoexfoliation syndrome, Amyloid-β, Aqueous humor, Visual field loss, Diseases, Medical research, Neurology, Neuroscience

## Abstract

Amyloid-β (Aβ) accumulation is a well-established cause of neurotoxicity in the brain and a major factor in Alzheimer’s disease. However, its role in ocular neurodegeneration remains unclear. In this prospective study, we evaluated the correlation between aqueous humor Aβ concentrations and retinal nerve fiber layer (RNFL) thickness in patients with open-angle glaucoma (including primary open-angle glaucoma and normal-tension glaucoma) and exfoliation glaucoma. A total of 154 patients who underwent either cataract or glaucoma surgery were included and categorized into four groups—cataract (*n* = 90), glaucoma (*n* = 27), pseudoexfoliation syndrome (*n* = 21), and exfoliation glaucoma (*n* = 16). Aβ concentrations in aqueous humor samples collected during surgery were measured using enzyme-linked immunosorbent assay. The mean Aβ1–40 and Aβ1–42 concentrations were significantly higher in exfoliation glaucoma and pseudoexfoliation syndrome groups than those in cataract group. Aβ1–40 and Aβ1–42 concentrations were strongly and positively correlated across all groups. Aβ concentrations were negatively correlated with RNFL thickness in glaucoma and exfoliation glaucoma groups. These findings indicate a potential role for soluble Aβ peptides in the pathophysiology and progression of glaucoma.

## Introduction

Primary open-angle glaucoma (POAG) is the most common type of glaucoma and is characterized by the degeneration of retinal ganglion cells (RGCs) and thinning of the retinal nerve fiber layer (RNFL), typically triggered by elevated intraocular pressure (IOP). These changes lead to progressive visual field loss and eventual blindness^1–3^. Since elevated IOP is the primary risk factor for POAG, current treatment strategies focus on lowering IOP. However, in certain types of glaucoma—such as normal tension glaucoma (NTG) and exfoliation glaucoma—visual field deterioration can progress even when IOP remains within the normal range (≤ 21 mmHg)^4^. This suggests that IOP-independent mechanisms may contribute to RGC degeneration. Alzheimer’s disease (AD), which affects approximately 50 million people worldwide, is characterized by the accumulation of neurotoxic amyloid-β (Aβ) peptides—derived from amyloid precursor protein (APP)—and the formation of neurofibrillary tangles in the brain, resulting in cognitive decline and brain atrophy^5^. Both glaucoma and AD are progressive neurodegenerative disorders, and numerous studies have reported potential links between them. Emerging evidence indicates that patients with AD are more likely to develop glaucoma, which is similarly marked by RGC loss and RNFL thinning^6–8^.

According to the original amyloid hypothesis, the primary cause of AD is the accumulation and deposition of neurotoxic Aβ peptides in the brain^9,10^. In contrast, the more recent oligomer hypothesis proposes that soluble Aβ oligomers—the most neurotoxic form of Aβ—disrupt synaptic plasticity and cause cellular degeneration and death^11^. Deposition of Aβ oligomers has been observed in the retinas of postmortem eyes from patients with AD^12^, and similarly, Aβ puncta have been detected in the retinas of postmortem eyes from patients with glaucoma^13^. These findings suggest a potential association between optic neuropathy in AD and glaucoma.

While Aβ is a hallmark of AD, only a few studies have examined the relationship between Aβ concentrations in the anterior aqueous humor^14,15^ and glaucoma-related changes in RNFL thickness^15^. Even fewer investigations have focused on Aβ levels in patients with pseudoexfoliation syndrome^16^, a systemic condition characterized by the accumulation of white, flaky fibrillar extracellular material in ocular tissues. Pseudoexfoliation syndrome affects approximately 25% of patients with POAG worldwide^17^ and can progress to exfoliation glaucoma due to impaired aqueous humor outflow caused by exfoliative material, leading to elevated IOP and RGC loss^17,18^.

Although accumulated Aβ is known to exert neurotoxic effects in the brain^9–11^, its role in ocular neurodegeneration remains poorly understood. We aimed to assess whether aqueous humor Aβ concentrations are associated with RNFL thinning in glaucoma and to explore a potential link between glaucoma and AD.

## Results

**Fig. 1:**
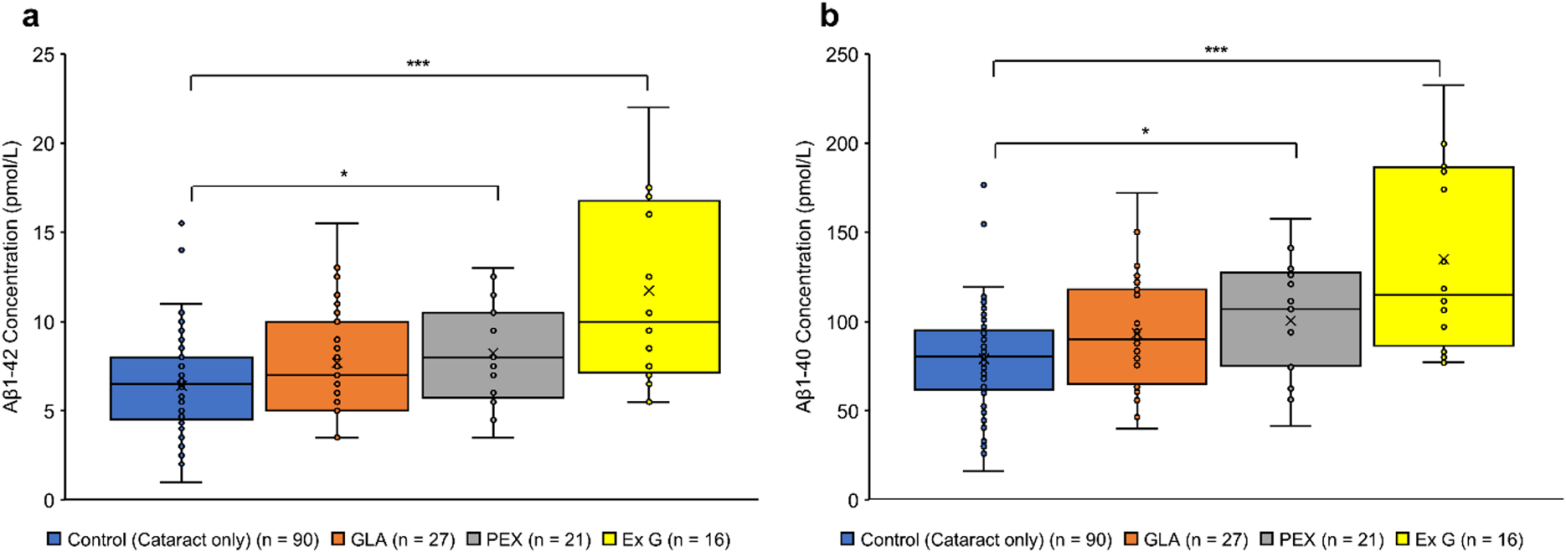
Aβ concentrations across all groups. (**a**) Aβ1–42 concentration. (**b**) Aβ1–40 concentration. Box-and-whisker plots demonstrate the interquartile range (IQR), with the median indicated by a line inside the box and the mean by a “×”. Whiskers represent the minimum and maximum values within 1.5 × IQR of the quartiles. *P < 0.05 and ***P < 0.001 (Kruskal–Wallis test followed by Steel’s test). Aβ, Amyloid-β; GLA, glaucoma; PEX, pseudoexfoliation syndrome; Ex G, exfoliation glaucoma.

**Fig. 2:**
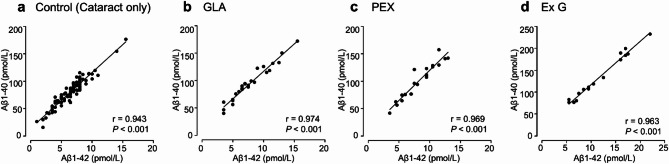
Scatterplots showing the correlation between Aβ1–42 and Aβ1–40 concentrations in Group Control (**a**), Group GLA (**b**), Group PEX (**c**), and Group Ex G (**d**). Correlations are calculated using Spearman’s rank correlation coefficient. Aβ, Amyloid-β; GLA, glaucoma; PEX, pseudoexfoliation syndrome; Ex G, exfoliation glaucoma.

**Fig. 3:**
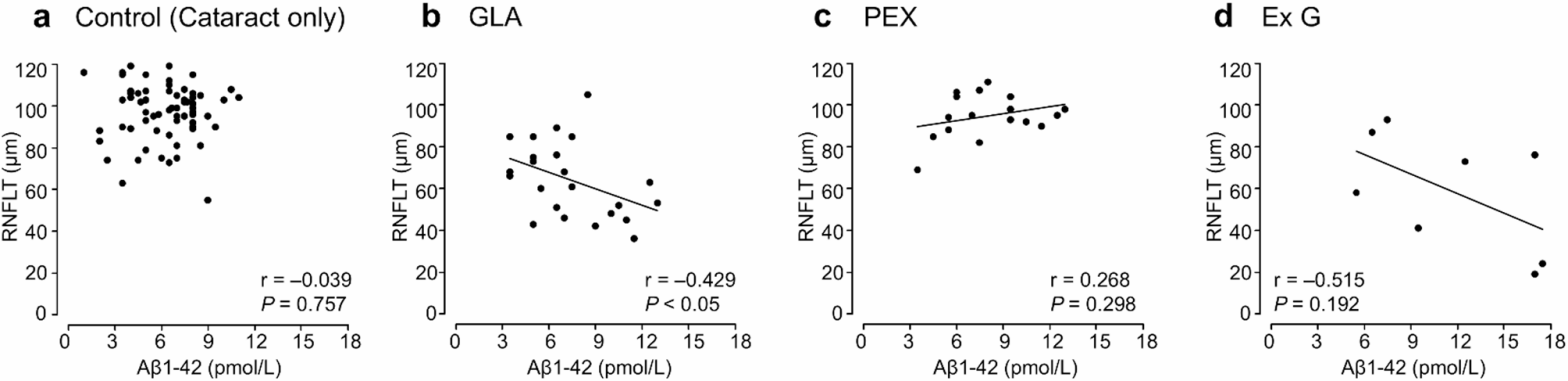
Scatterplots showing the correlation between Aβ1–40 concentration and RNFLT in Group Control (**a**), Group GLA (**b**), Group PEX (**c**), and Group Ex G (**d**). Correlations are calculated using Spearman’s rank correlation coefficient. Aβ, Amyloid-β; GLA, glaucoma; PEX, pseudoexfoliation syndrome; Ex G, exfoliation glaucoma; RNFLT, retinal nerve fiber layer thickness.

**Fig. 4:**
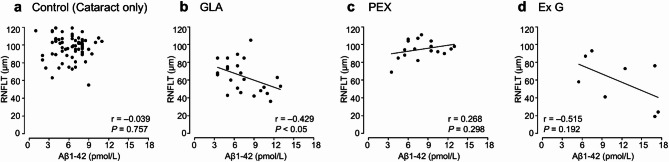
Scatterplots showing the correlation between Aβ1–42 concentration and RNFLT in Group Control (**a**), Group GLA (**b**), Group PEX (**c**), and Group Ex G (**d**). Correlations are calculated using Spearman’s rank correlation coefficient. Aβ, Amyloid-β; GLA, glaucoma; PEX, pseudoexfoliation syndrome; Ex G, exfoliation glaucoma; RNFLT, retinal nerve fiber layer thickness.

Table 1 presents the patients’ characteristics and results. No significant differences were observed in the sex ratio among the four groups. The cataract group will serve as the control group, referred to as “Control” from this point forward. The mean age was significantly higher in Group Exfoliation Glaucoma (Ex G) (81.19 ± 4.60 years) than in Group Control (Cataract only) (73.21 ± 8.24 years) (*P* < 0.001). The mean concentrations of Aβ1–40 and Aβ1–42 were significantly higher in Group Ex G (134.88 ± 49.72 pmol/L and 11.75 ± 4.99 pmol/L, respectively; *P* < 0.001) and Group Pseudoexfoliation Syndrome (PEX) (100.59 ± 31.46 pmol/L and 8.25 ± 2.72 pmol/L, respectively; *P* < 0.05) than in Group Control (79.17 ± 25.74 pmol/L and 6.40 ± 2.41 pmol/L, respectively) (Fig. 1). A very strong and statistically significant positive correlation was observed between Aβ1–40 and Aβ1–42 concentrations across all groups (*r* > 0.9; *P* < 0.001) (Fig. 2). The mean total RNFL thickness was significantly lower in Group GLA (64.13 ± 17.60 μm) and Group Ex G (58.88 ± 26.40 μm) than in Group Control (96.86 ± 13.25 μm) (*P* < 0.001). Aβ1–40 and Aβ1–42 concentrations showed moderate negative correlations with RNFL thickness in Group GLA (*r* = − 0.501; *P* < 0.05, *r* = − 0.429; *P* < 0.05, respectively) and in Group Ex G (*r* = − 0.395; not significant, *r* = − 0.515; not significant, respectively) (Figs. 3 and 4). Neither Aβ1–40 nor Aβ1–42 concentrations were correlated with age (*r* = − 0.080 and *r* = − 0.123, respectively). The mean preoperative IOP was significantly higher in Group Ex G (19.10 ± 7.37 mmHg) than in Group Control (13.57 ± 2.77 mmHg) (*P* < 0.01). Neither Aβ1–40 nor Aβ1–42 concentrations were correlated with the preoperative IOP (*r* = 0.104 and *r* = 0.147, respectively).

**Table 1:** Patients’ characteristics and results. Data are presented as mean ± SD.

Variable	Control (*n* = 90)	GLA (*n* = 27)		PEX (*n* = 21)		Ex G (*n* = 16)	
	Mean ± SD	Mean ± SD	*P*	Mean ± SD	*P*	Mean ± SD	*P*
Age	73.21 ± 8.24	74.48 ± 6.44	0.871	76.00 ± 7.14	0.430	81.19 ± 4.60***	< 0.001
Male/Female	37/53	12/15		5/16		7/9	0.443
Aβ1–42 (pmol/L)	6.40 ± 2.41	7.69 ± 3.03	0.252	8.25 ± 2.72*	0.023	11.75 ± 4.99***	< 0.001
Aβ1–40 (pmol/L)	79.17 ± 25.74	93.61 ± 31.09	0.105	100.59 ± 31.46*	0.018	134.88 ± 49.72***	< 0.001
Aβ1–42/1–40 ratio	0.080 ± 0.011	0.081 ± 0.008	0.919	0.082 ± 0.007	0.529	0.085 ± 0.008	0.062
Pre-op IOP (mmHg)	13.57 ± 2.77	13.73 ± 3.05	0.997	14.65 ± 3.84	0.863	19.10 ± 7.37**	< 0.01
RNFLT (µm)	96.86 ± 13.25 (*n* = 66)	64.13 ± 17.60*** (*n* = 23)	< 0.001	94.76 ± 10.10 (*n* = 18)	0.738	58.88 ± 26.40*** (*n* = 8)	< 0.001
MD (dB)		−8.88 ± 6.81 (*n* = 21)				−14.68 ± 10.22 (*n* = 8)	

*P < 0.05, **P < 0.01 and ***P < 0.001 (compared with that of the control group). GLA = Glaucoma; PEX = Pseudoexfoliation syndrome; Ex G = Exfoliation glaucoma; SD = standard deviation; Aβ = amyloid-β; pre-op IOP = preoperative intraocular pressure; RNFLT = retinal nerve fiber layer thickness; MD = mean deviation; dB = decibels.

## Discussion

Previous studies have evaluated Aβ concentrations in cerebrospinal fluid (CSF), aqueous humor, and plasma^15,19,20^. According to a meta-analysis of these studies, the mean Aβ1–42 concentration in CSF was 554 pg/mL (range: 60–1777 pg/mL) in patients with AD and 979 pg/mL (range: 147–2400 pg/mL) in the control groups^19^. In plasma, the mean Aβ1–42 concentration was 71 pg/mL (range: 5–277 pg/mL) in patients with AD and 66 pg/mL (range: 6–195 pg/mL) in controls^20^. In the present study, the mean Aβ1–42 concentration in aqueous humor was 11.75 pmol/L (equivalent to 53.0 pg/mL) in patients with exfoliation glaucoma and 6.40 pmol/L (28.9 pg/mL) in patients with cataract only. Although research methodologies and mean values vary across studies, Aβ concentrations were consistently highest in CSF. The brain parenchyma, which accounts for approximately 80% of total brain volume, contains a high density of neuronal cells^21^. In contrast, although the retina is rich in neurons, it occupies only a small portion of the eye’s total volume^22^, which may account for the lower Aβ concentrations observed in the aqueous humor. In this study, patients with glaucoma, exfoliation glaucoma, or pseudoexfoliation syndrome exhibited higher aqueous humor Aβ concentrations than those with cataracts only. These findings are consistent with previous studies reporting slightly elevated Aβ1–40 and Aβ1–42 levels in the aqueous humor of patients with glaucoma or pseudoexfoliation syndrome^14–16^. A key distinction in our study is the identification of statistically significant differences in Aβ concentrations between groups. This discrepancy may be due to the smaller sample sizes in the study by Janciauskiene et al.^14^, or because the study by Cappelli et al.^15^ included glaucoma patients with thicker RNFLT than those in our study. Neither Lesiewska et al.^16^ nor our study considered the severity of pseudoexfoliation, which limits the ability to make direct comparisons. These results contrast with those in AD, where Aβ1–42 concentrations were significantly lower in the CSF of patients with AD, while Aβ1–40 concentrations remained comparable to those in controls^23–25^. This discrepancy may be explained by the aggregation and deposition of Aβ1–42 peptides as senile plaques in the brains of patients with AD, which limits their diffusion into the CSF. Similarly, plasma Aβ1–42 concentrations tend to be slightly lower in patients with AD, though the difference is not statistically significant, whereas Aβ1–40 levels remain largely comparable to those in cognitively normal individuals^20^. Jensen et al. reported that CSF Aβ1–42 concentrations may increase during the early stages of AD but decline as the disease progresses, due to increasing deposition of Aβ1–42 in brain tissue^26^. The pathophysiology of AD and glaucoma may share common features, particularly the involvement of abnormal Aβ metabolism. Both conditions may involve increased production of Aβ peptides—particularly Aβ1–42—followed by local accumulation and associated neurodegeneration in the brain and eye, respectively.

Consistent with a previous study reporting a strong positive correlation between Aβ1–40 and Aβ1–42 concentrations in the aqueous humor^27^, we observed a very strong and statistically significant positive correlation between these two peptides across all patient groups, regardless of the presence of glaucoma or pseudoexfoliation syndrome. The overall Aβ1–42/Aβ1–40 ratio was approximately 0.08, and although the difference was not statistically significant, it showed a tendency to be higher in patients with exfoliation glaucoma. These findings contrast with those observed in AD, where the correlation between Aβ1–40 and Aβ1–42 concentrations in the CSF is stronger in control groups than in patients with AD^28^, and the CSF Aβ1–42/Aβ1–40 ratio is significantly lower in patients with AD than in controls^23^. This discrepancy may suggest that Aβ1–42 deposition in the eye is less pronounced than in the brain, potentially due to differences in tissue composition and the site of neurodegeneration. In glaucoma, apoptosis primarily affects RGCs, which are located on the inner surface of the retina^12^. This localized degeneration may result in limited Aβ1–42 aggregation or plaque formation, leading to relatively higher detectable levels in the aqueous humor than those in the CSF of patients with AD.

In the central nervous system, Aβ is primarily synthesized by neurons and accumulates in the CSF. Mathieu et al. demonstrated that CSF enters the optic nerve via a glymphatic pathway, suggesting that this pathway may play a role in the pathophysiology of glaucoma^29^. Additionally, several studies have proposed that Aβ is also synthesized within the retina—mainly by RGCs and the retinal pigment epithelium—and subsequently secreted into the vitreous humor before being transported to the aqueous humor^30,31^. Based on this evidence, we speculate that Aβ originates from retinal synthesis or may be secreted from the plasma into the aqueous humor via the ciliary epithelium. Supporting this hypothesis, Prakasam et al. reported that in bovine eyes, Aβ concentrations in the vitreous humor were approximately twice as high as in the aqueous humor^30^. Similarly, Sampani et al. found that in human eyes, Aβ concentrations in the vitreous humor exceeded those in the plasma and aqueous humor^32^. Although Aβ concentrations in the vitreous humor were not measured in the present study, we speculate that Aβ may accumulate throughout the ocular compartments, particularly in patients with exfoliation glaucoma, where elevated intraocular Aβ concentrations were most prominent. Further research will be required to assess Aβ concentrations in both the aqueous and vitreous humor by collecting samples simultaneously from the same eyes.

Our study demonstrated significant RNFL thinning in patients with glaucoma or exfoliation glaucoma, alongside negative correlations between Aβ concentrations and RNFL thickness. These findings may suggest a possible link between neurotoxic Aβ and the progression of glaucoma, although it remains unclear whether Aβ accumulation is a cause or a consequence of the disease. Guo et al. evaluated the association between Aβ deposition and RGC apoptosis using an experimental glaucoma rat model^33^. They reported that Aβ deposition in the RGC layer significantly increased following IOP elevation, and intravitreally injected Aβ1–42 oligomer (0.55 nmol) induced significantly more RGC apoptosis than Aβ25–35. In our study, Aβ1–42 concentration in patients with glaucoma, pseudoexfoliation syndrome, or exfoliation glaucoma ranged from 3.5 pmol/L to 22.0 pmol/L, corresponding to approximately 0.35 nmol to 2.20 nmol in the 0.1 mL of aqueous humor samples collected—comparable to or exceeding the dosage used in the experimental model. This amount is roughly equivalent to that injected into glaucoma rat models described by Guo et al., suggesting that the Aβ1–42 concentrations observed in the aqueous humor of patients with glaucoma, pseudoexfoliation syndrome, or exfoliation glaucoma in our study may be sufficient to induce RGC apoptosis. This, in turn, could contribute to RNFL thinning. McKinnon et al. reported that elevated IOP activated caspase pathways and led to abnormal processing of APP in RGCs in ocular hypertension rat models^34,35^. In our study, preoperative IOP was significantly higher in patients with exfoliation glaucoma. This finding suggests that elevated IOP may induce Aβ overexpression, disrupting the balance between Aβ synthesis and clearance and resulting in its accumulation and subsequent RGC loss. We further speculate that obstruction of aqueous humor outflow by exfoliative material could exacerbate Aβ accumulation, causing persistent neurodegeneration even after IOP is lowered.

This study has some limitations. First, the relatively small sample size of patients with glaucoma and pseudoexfoliation syndrome may limit the generalizability of the findings. Second, patients with exfoliation glaucoma were significantly older than those in other groups; however, there was no correlation between age and Aβ concentrations. Third, detailed pre-treatment information, particularly regarding IOP prior to the initiation of IOP-lowering medications, was unavailable for most glaucoma patients, as they were referred from other clinics. Fourth, the use of IOP-lowering eye drops in nearly all patients with glaucoma likely suppressed aqueous humor production and reduced its circulation and outflow, potentially affecting the measured concentrations of Aβ. Fifth, this study focused solely on Aβ, even though both glaucoma and pseudoexfoliation syndrome are multifactorial diseases with complex pathophysiology. Although previous studies have conducted proteomic analyses to identify a broader spectrum of molecular changes^36–38^, the precise mechanisms underlying these conditions remain unclear. To address these limitations, we are planning future studies to investigate proteomic alterations in both aqueous and vitreous humor.

In conclusion, aqueous humor Aβ concentrations were particularly elevated in patients with exfoliation glaucoma and showed negative correlations with RNFL thickness. These findings suggest a potential role of soluble Aβ peptides in the pathogenesis and progression of glaucoma, possibly involving mechanisms that partially overlap with those implicated in AD.

## Methods

### Patients

This prospective, comparative study included 127 eyes from 127 patients who underwent cataract surgery and 27 eyes from 27 patients who underwent glaucoma surgery at Ohashi Eye Center or Caress Sapporo Tokeidai Memorial Hospital, Sapporo, Japan, between October 2022 and May 2025. Patients were eligible for inclusion if they had no ocular pathology other than cataract, glaucoma (including POAG and NTG), pseudoexfoliation syndrome, or exfoliation glaucoma. Patients with systemic conditions, such as diabetes mellitus or high myopia (≥ − 6.00 diopters), were excluded. Patients were divided into four groups: 27 with glaucoma, including POAG and NTG (Group GLA); 21 with pseudoexfoliation syndrome (Group PEX); 16 with exfoliation glaucoma (Group Ex G); and 90 with cataract only (Group Control).

Glaucoma was diagnosed using swept-source optical coherence tomography (SS-OCT) (DRI OCT Triton, TOPCON CORPORATION, Tokyo, Japan) and the Humphrey Field Analyzer (HFA III, Carl Zeiss Meditec AG, Jena, Germany) and defined based on optic nerve and visual field changes, specifically with a mean deviation (MD) of less than 0 decibels (dB). Patients with an SS-OCT image quality factor below 45 or a fixation loss of 20% or more on the HFA were excluded from the RNFL thickness and MD analyses, as well as from correlation analysis. Among patients with glaucoma and exfoliation glaucoma, 18 underwent combined trabeculotomy and cataract surgery, 7 underwent combined iStent inject implantation and cataract surgery, 3 underwent combined Preserflo Microshunt implantation and cataract surgery, 2 underwent trabeculotomy alone, 1 underwent combined trabeculectomy and cataract surgery, and 1 underwent Ex-Press removal followed by trabeculectomy. A total of 43 patients received IOP-lowering eye drops, including carteolol hydrochloride, ripasudil hydrochloride hydrate, and pilocarpine hydrochloride.

Pseudoexfoliation was diagnosed by slit-lamp examination (SL130, Carl Zeiss Meditec AG, Jena, Germany) and defined by the presence of exfoliative material on the anterior lens surface, pupillary border, or iris surface.

This study was conducted in accordance with the tenets of the Declaration of Helsinki and approved by the Institutional Review Board of Nagoya Eye Clinic (UMIN Clinical Trials Registry ID: UMIN000058049). Informed consent was obtained from all patients prior to the collection of aqueous humor samples.

### Methods for collection of anterior aqueous humor samples

Approximately 0.1 mL of anterior aqueous humor was collected with a 30-gauge syringe through the surgical incision at the start of cataract or glaucoma surgery. Immediately after collection, the samples were transferred to gas-sterilized plastic tubes (sterilized for 24 h) and stored at − 80 °C until analysis. Concentrations of Aβ1–40 and Aβ1–42 were measured using sensitive and specific enzyme-linked immunosorbent assay (ELISA), according to the manufacturer’s instructions: Human β-Amyloid (1–40) ELISA Kit Wako II (298–64601) and Human β-Amyloid (1–42) ELISA Kit Wako, High Sensitive (296–64401) (Fujifilm Wako Pure Chemical Corporation, Osaka, Japan). The minimum detectable concentrations were 1.0 pmol/L for Aβ1–40 and 0.1 pmol/L for Aβ1–42. IOP and RNFL thickness were measured preoperatively in all patients, and the degree of visual field loss was assessed in patients with glaucoma. Correlations between Aβ1–40 and Aβ1–42 concentrations, and RNFL thickness were subsequently evaluated.

### Statistical analysis

All statistical analyses were performed using EZR^39^ (Saitama Medical Center, Jichi Medical University, Saitama, Japan), a graphical user interface for R (The R Foundation for Statistical Computing, Vienna, Austria). EZR is a modified version of R Commander that incorporates statistical functions commonly used in biostatistics. The Kolmogorov–Smirnov test or Shapiro–Wilk test was used to assess the normality of the data distribution. One-way analysis of variance (ANOVA), followed by Tukey’s post hoc test, was used to compare mean age across groups. The Kruskal–Wallis test, followed by Steel’s test, was employed to assess differences in Aβ1–40 and Aβ1–42 concentrations, the Aβ1–42/Aβ1–40 ratio, RNFL thickness, visual field loss (MD), and preoperative IOP between the Group Control and the other three groups. Fisher’s exact test was used to compare male-to-female ratios among groups. Spearman’s rank correlation coefficient was applied to evaluate correlations between Aβ1–40 and Aβ1–42, Aβ1–40 and RNFL thickness, Aβ1–42 and RNFL thickness, Aβ1–40 and age, Aβ1–42 and age, Aβ1–40 and preoperative IOP, and Aβ1–42 and preoperative IOP. A *P*-value of less than 0.05 was considered statistically significant.

## Data Availability

The datasets generated and/or analyzed during the current study are available from the corresponding author on reasonable request.
